# Supplementary material for: Bispecific BCMA/CD24 CAR-T cells control multiple myeloma growth
Source: Nat Commun. 2024 Jan 19;15:615. doi: 10.1038/s41467-024-44873-4 (PMC10798961; doi:10.1038/s41467-024-44873-4)
Supplement: Supplementary file 1 — Supplementary Information [file 41467_2024_44873_MOESM1_ESM.pdf]

Supplementary information

Bispecific BCMA/CD24 CAR-T Cells Control Multiple Myeloma Growth

Authors

Fumou Sun,[1,†] Yan Cheng,[1,†] Visanu Wanchai,[1] Wancheng Guo,[1] David Mery,[1] Hongwei Xu,[1] Dongzheng Gai,[1] Eric Siegel,[2] Clyde Bailey,[1] Cody Ashby,[3] Samer Al Hadidi,[1] Carolina Schinke,[1] Sharmilan Thanendrarajan,[1] Yupo Ma,[4] Qing Yi,[5] Robert Orlowski,[6] Maurizio Zangari,[1] Frits van Rhee,[1] Siegfried Janz,[7] Gail Bishop,[8] Guido Tricot,[1] John D Shaughnessy Jr,[1] Fenghuang Zhan[1,*]

[1] Myeloma Center, Winthrop P. Rockefeller Institute, Department of Internal Medicine, University of Arkansas for Medical Sciences, Little Rock, AR 72205, USA.

[2] Department of Biostatistics, University of Arkansas for Medical Sciences, Little Rock, AR 72205, USA.

[3] Department of Biomedical Informatics, University of Arkansas for Medical Sciences, Little Rock, AR 72205, USA.

[4] iCell Gene Therapeutics LLC, Research & Development Division, Stony Brook, NY 11790, USA.

[5] Center for Translational Research in Hematologic Malignancies, Houston Methodist Cancer Center, Houston Methodist Research Institute, Houston, TX 77030, USA.

[6] Department of Lymphoma and Myeloma, The University of Texas MD Anderson Cancer Center, Houston, TX 77030, USA.

[7] Division of Hematology and Oncology, Department of Medicine, Medical College of Wisconsin, Milwaukee, WI 53226, USA.

[8] Department of Microbiology and Immunology, University of Iowa and VA Medical Center, Iowa City, IA 52242, USA.

[†] These authors contributed equally: Fumou Sun, Yan Cheng.

* Corresponding Author: Fenghuang Zhan, Myeloma Center, Winthrop P. Rockefeller Institute, Department of Internal Medicine, University of Arkansas for Medical Sciences, 4301 W. Markham St., Slot # 508, Little Rock, Arkansas 72205, USA. Tel: 501-526-6000 ext. 25228. Email: FZhan@uams.edu

Supplementary Figure 1.

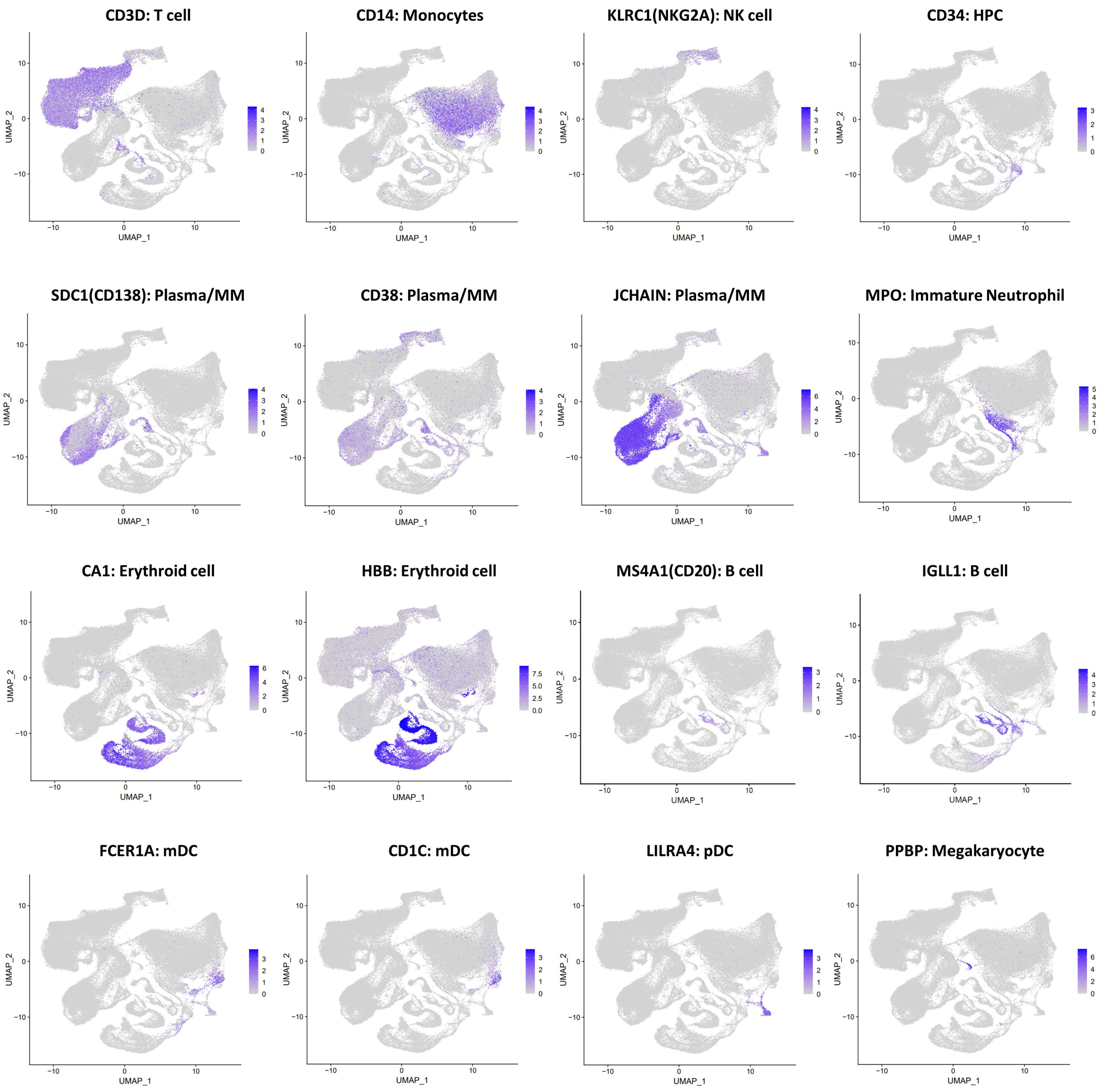

Supplementary Figure 1. Marker genes demonstrating cell type identity of clusters. UMAP representation of cell populations: T cell, Monocyte, Plasma/MM, B cell, Erythroid cell, Immature Neutrophils, Myeloid dendritic cell (mDC), Plasmacytoid dendritic cell (pDC), Hematopoietic progenitor cell (HPC) and Megakaryocyte. In each subplot, cells are colored by log-normalized expression values for a given cell type specific marker gene.

Supplementary Figure 2.

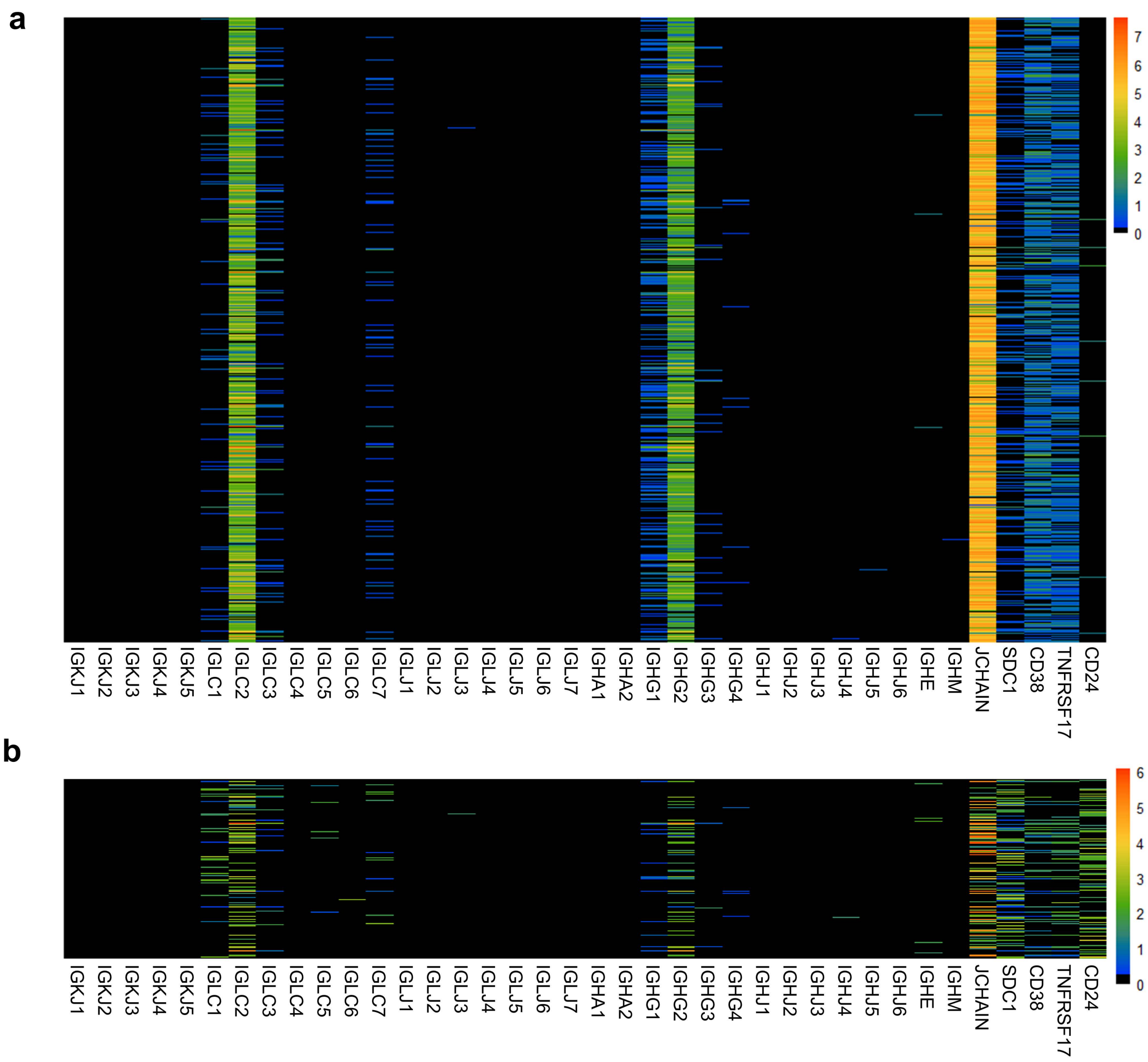

Supplementary Figure 2. Heatmap representing the expression level of the immunoglobulins, JCHAIN, SDC1, CD38, TNFRSF17 and CD24 in Plasma/MM cells (Patient #19). (a) Heatmap of Plasma/MM cells of pre-infusion from patient #19. **(b)** Heatmap of Plasma/MM cells of post-infusion day 28 from patient #19. Each row represents an individual Plasma/MM cell, and each column represents a gene. (red: high express; black: no express). The heatmap showed that patient #19 had an IgGλ MM. The proportion and intensity of CD24-positive cells was clearly increased in residual MM cells.

Supplementary Figure 3.

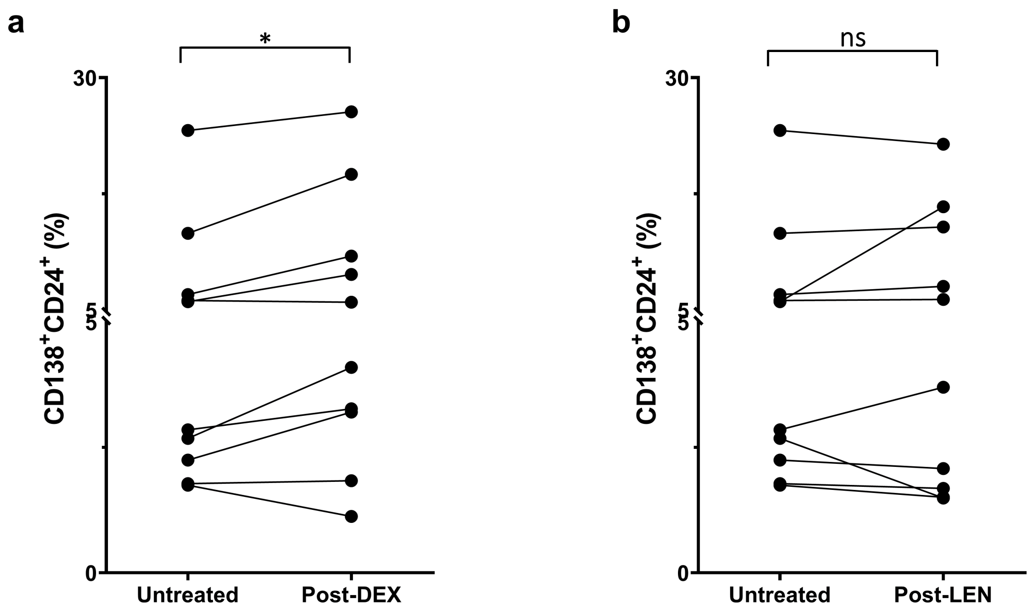

Supplementary Figure 3. The frequency of CD138⁺CD24⁺ cells after dexamethasone or lenalidomide treatment. (a) Flow cytometry analysis of patient samples and the association between drug response and CD24 expression in MM cells (n =10). The frequency of the subpopulation of $CD138^{+}CD24^{+}$ cells increase in 8 of 10 primary myeloma samples post-dexamethasone (DEX) treatment. **(b)** The frequency of $CD138^{+}CD24^{+}$ cells didn't change significantly in BMMC samples post-lenalidomide (LEN) (n =10). Paired t-test was used. All tests are two-sided. Data are presented as mean values +/- SD. $*P < .05$, ns = $P > .05$. Raw data is provided in the Source Data file. Exact *P* values for each comparison can be found in Supplementary Data 1.

Supplementary Figure 4.

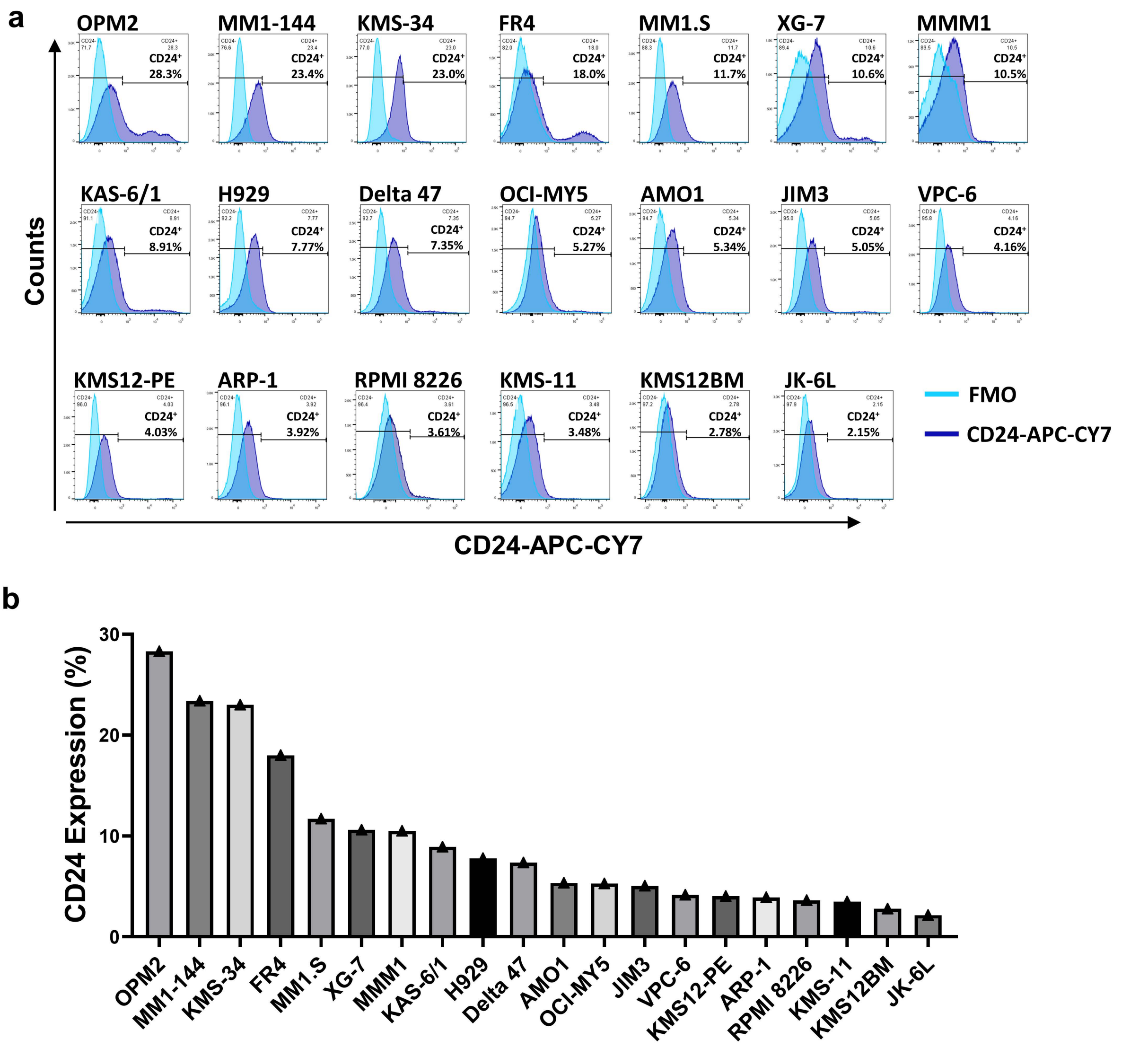

Supplementary Figure 4. CD24 expression in MM cell lines. (a) Flow cytometry analysis of CD24 expression in 20 MM cell lines. All the MM cell lines expressed CD24. Some cell lines highly expressed CD24, such as OPM2, MM1-144, and KMS-34. Fluorescence minus one (FMO) control were samples stained with all of the fluorophores, minus CD24-APC-CY7. **(b)** Bar plot showing expression levels of CD24 in 20 MM cell lines. Raw data is provided in the Source Data file.

Supplementary Figure 5.

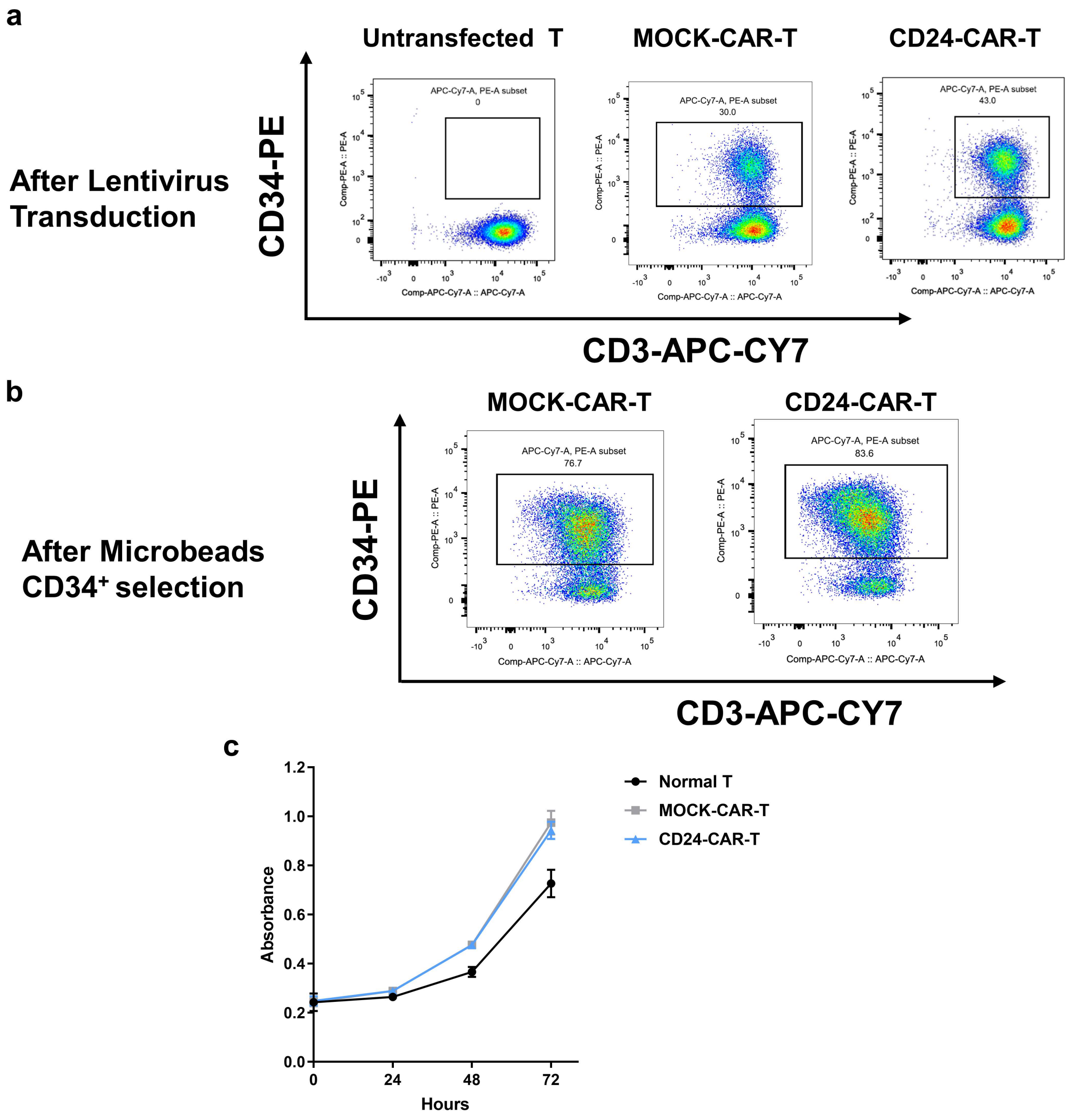

Supplementary Figure 5. Generation of CD24-CAR-T cells. (a) Lentivirus combined with the RetroNectin transduction technology was used to modify the $CD3^+$ T cells from healthy donor peripheral blood mononuclear cells (PBMCs). CAR-T cells were detected with flow cytometry and the RQR8-specific CD34 antibody. The CD34 positive rates were 43% for CD24-CAR-T cells. **(b)** CD34 Microbeads were used to isolate the CAR-T cells. After isolation, the CD34 positive rate increased to 83.6% for CD24-CAR-T cells. **(c)** CCK8 kits were used to detect CAR-T cell proliferation (n = 3 independent experiments). Raw data is provided in the Source Data file.

Supplementary Figure 6.

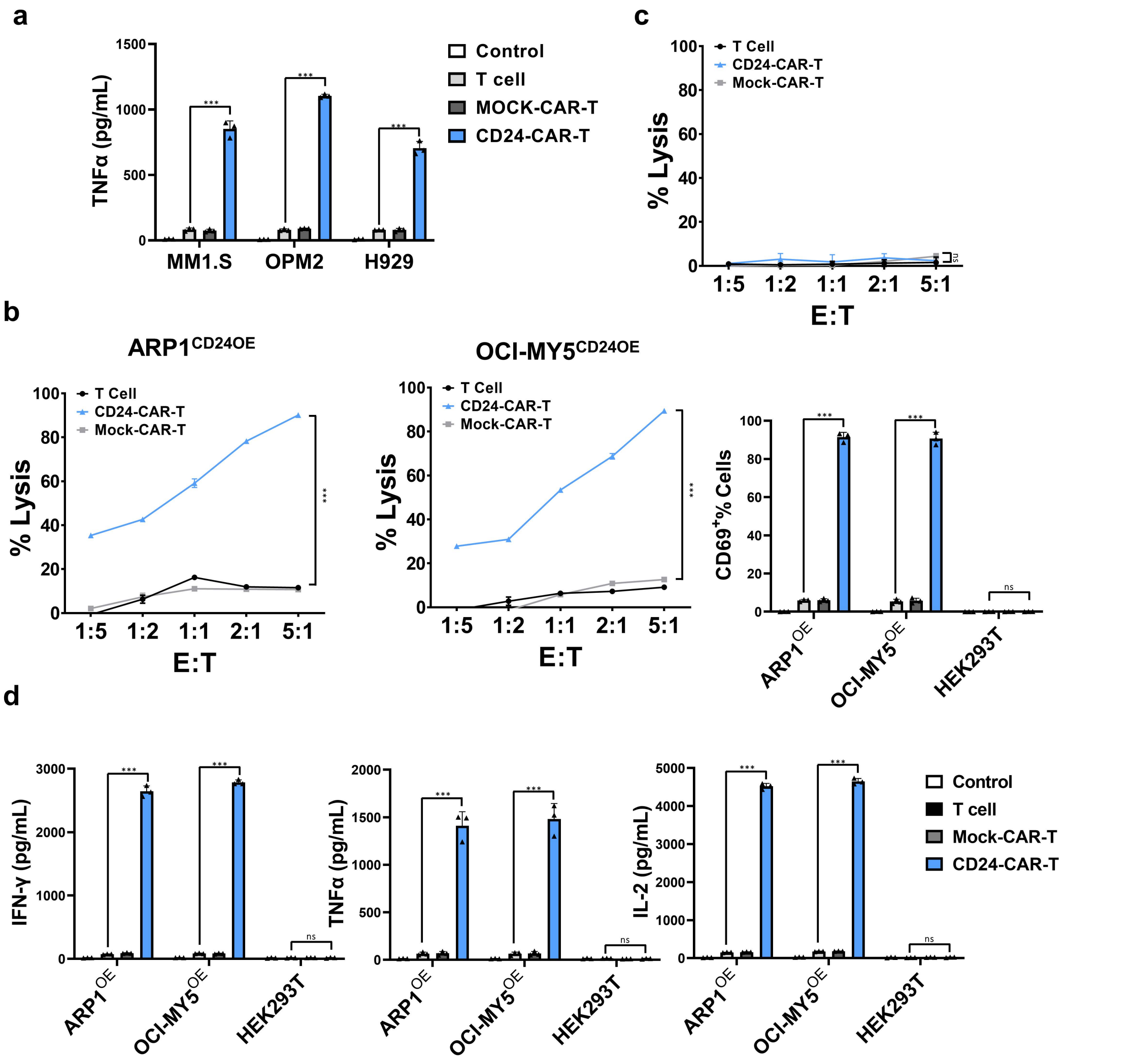

Supplementary Figure 6. CD24-CAR-T cells eliminate CD24-overexpress MM cells in vitro. (a) TNF-α concentrations on supernatants were detected when the E/T ratio was 5:1 after 24 hours of coculture (n = 3 independent experiments). **(b-c)** CAR-T or T cells were added to CD24-overexpress MM cell lines: ARP1^{CD24OE} and OCI-MY5^{CD24OE} or CD24-negtive human embryonic kidney cell line: HEK293 at the effector/target (E/T) ratio from 1:5 to 5:1. After 24 hours of coculture, cytolytic activity was measured (n = 3 independent experiments). **(d)** CD69 expression, IFN-γ, TNF-α and IL-2 concentrations was detected at the E/T ratio was 5:1 after 24 hours of coculture (n = 3 independent experiments). One-way ANOVA was used for statistical analysis. Data are presented as mean values +/- SD. ∗∗∗P < .001. ns = P > .05. Raw data is provided in the Source Data file. Exact P values for each comparison can be found in Supplementary Data 1.

Supplementary Figure 7

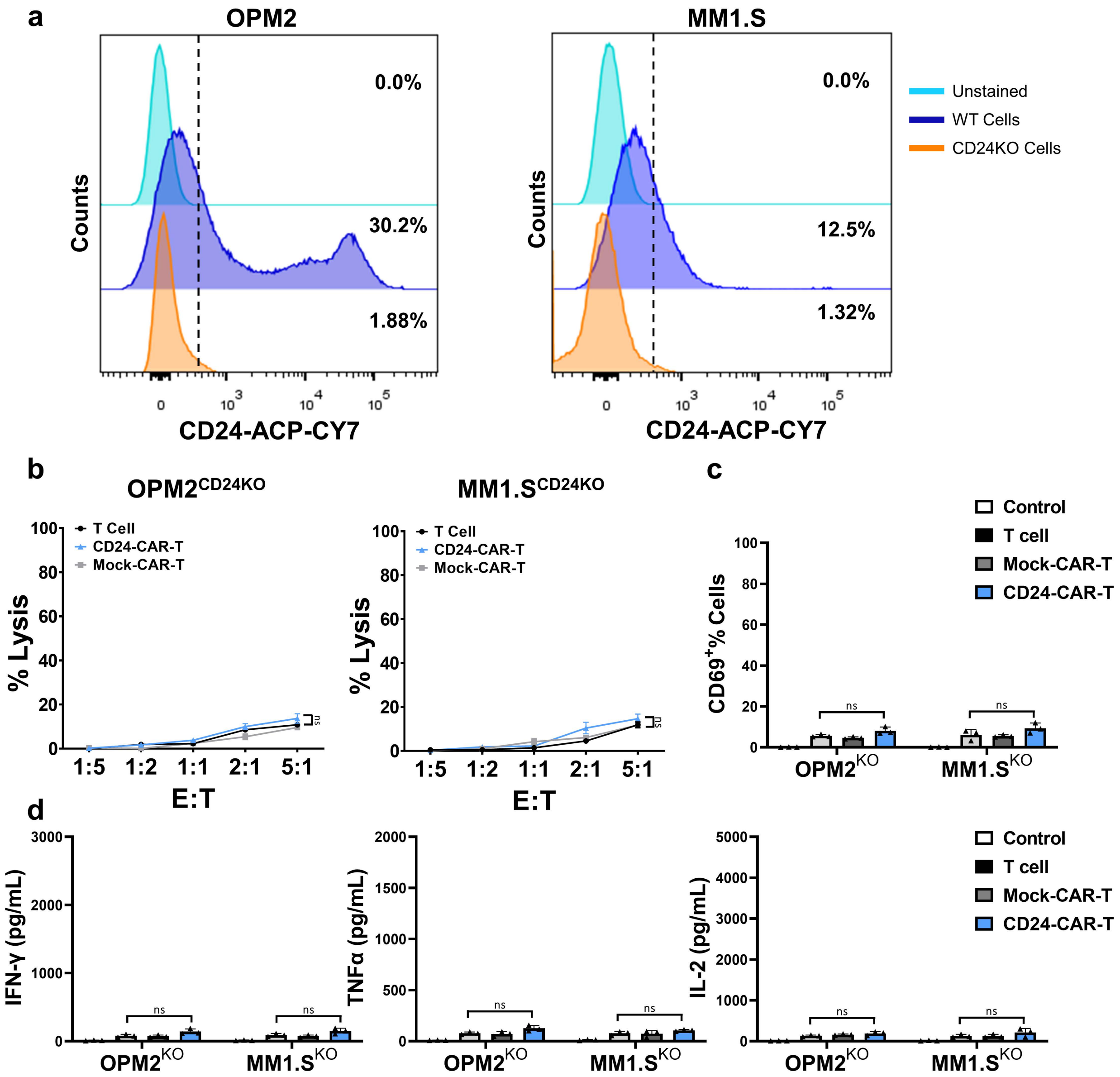

Supplementary Figure 7. CD24-CAR-T cells can't eliminate CD24-knockout MM cells in vitro. (a) Flow cytometry analysis of CD24 expression in CD24-knockout (CD24KO) MM cells. **(b)** CAR-T or T cells were added to CD24KO MM cell lines: $OPM2^{CD24KO}$ and $MM1.S^{CD24KO}$ at the effector/target (E/T) ratio from 1:5 to 5:1. After 24 hours of coculture, cytolytic activity was measured (n = 3 independent experiments). **(c-d)** CD69 expression, IFN-γ, TNF-α and IL-2 concentrations was detected at the E/T ratio was 5:1 after 24 hours of coculture (n = 3 independent experiments). One-way ANOVA was used for statistical analysis. Data are presented as mean values +/- SD. ns = $P > .05$. Raw data is provided in the Source Data file. Exact *P* values for each comparison can be found in Supplementary Data 1.

Supplementary Figure 8.

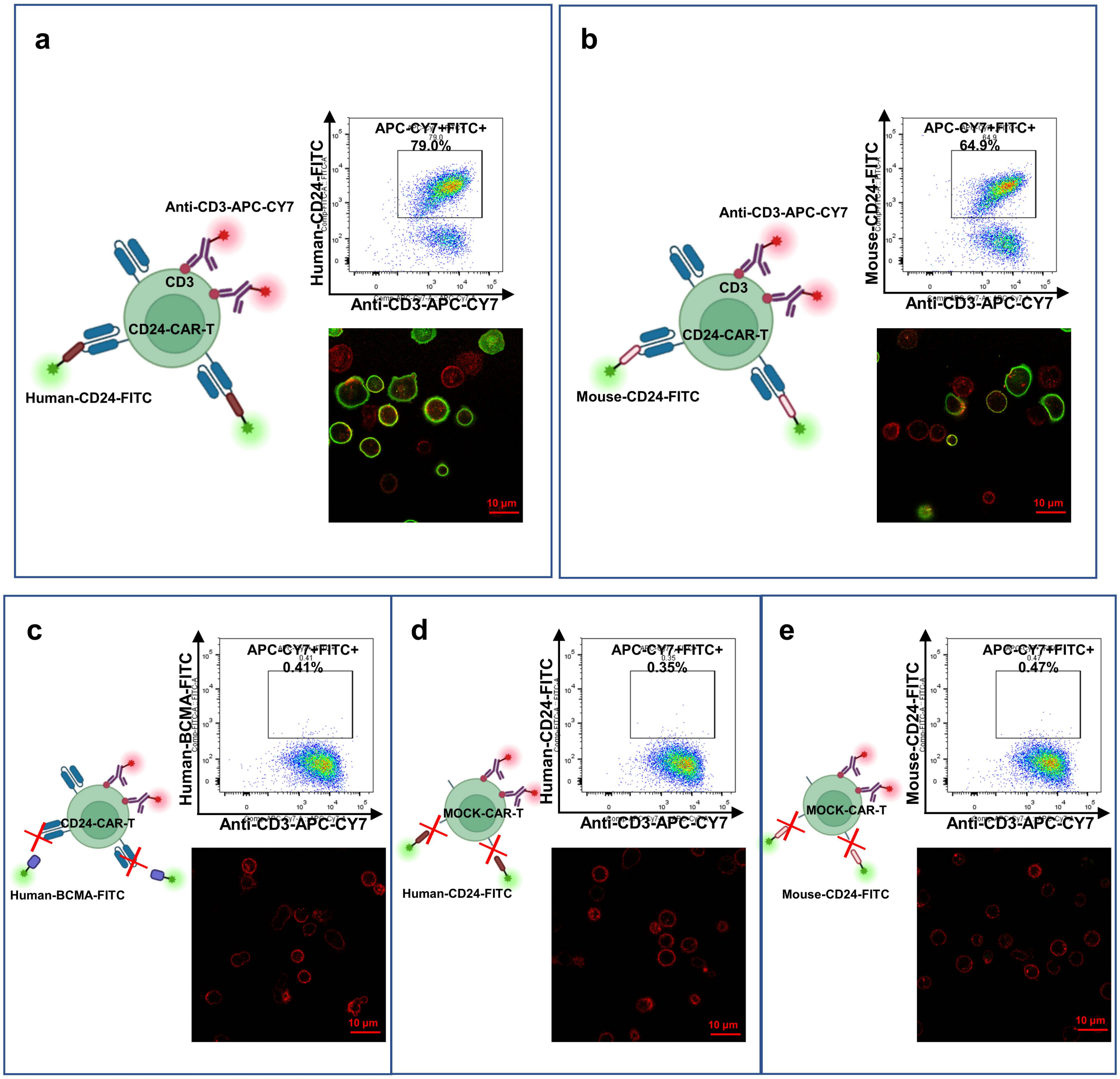

Supplementary Figure 8. Specificity and cross-reactivity assay. (a) FITC-Labeled Human CD24 (green) was used to evaluate the binding activity of CD24-CAR-T (red) with human CD24. **(b)** FITC-Labeled Mouse CD24 (green) was used to evaluate the binding activity of CD24-CAR-T (red) with mouse CD24. **(c)** FITC-Labeled Human BCMA (green) was used as negative control. **(d)** FITC-Labeled Human CD24 (green) was used to evaluate the binding activity of MOCK-CAR-T (red) with human CD24 or mouse CD24 **(e).** Flow cytometry and fluorescent images were used to detect the fluorescent light signals. The experiments of all panels were repeated twice with the same results.

Supplementary Figure 9.

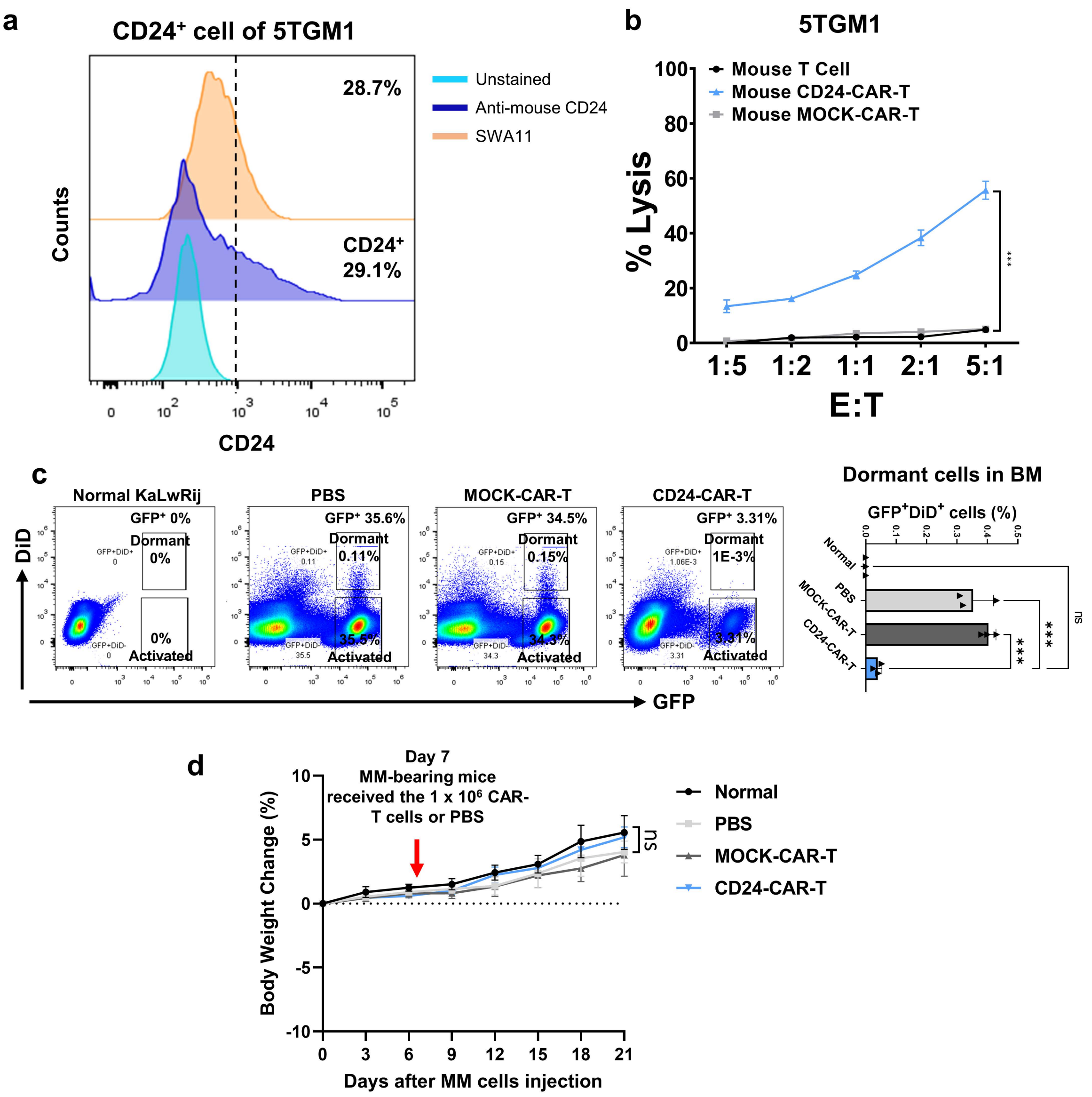

Supplementary Figure 9. CD24-CAR-T cells eliminate MM cells in vivo. (a) Flow cytometry analysis of CD24 expression in 5TGM1 cell line by anti-mouse CD24 antibody (dark blue). The SWA11 antibody could target the CD24 in 5TGM1 cells (yellow). **(b)** Mouse CAR-T or mouse T cells were added to 5TGM1 cells at the effector/target (E/T) ratio from 1:5 to 5:1. After 24 hours of coculture, cytolytic activity was measured (n = 3 independent experiments). **(c)** Representative $GFP^{+}DiD^{Hi}$ and $GFP^{+}DiD^{Neg}$ gating strategy to identify dormant and activated MM cell populations in 5TGM1 BM samples. Bar plot showing the percentage of dormant cells in BM (right) (n = 5 per group). **(d)** Mouse weight normalized to day 0 for each group, from the day after 5TGM1 MM cells injection (n = 5 per group). One-way ANOVA was used for statistical analysis. Data are presented as mean values +/- SD. ***P < .001, ns = P > .05. Raw data is provided in the Source Data file. Exact P values for each comparison can be found in Supplementary Data 1.

Supplementary Figure 10

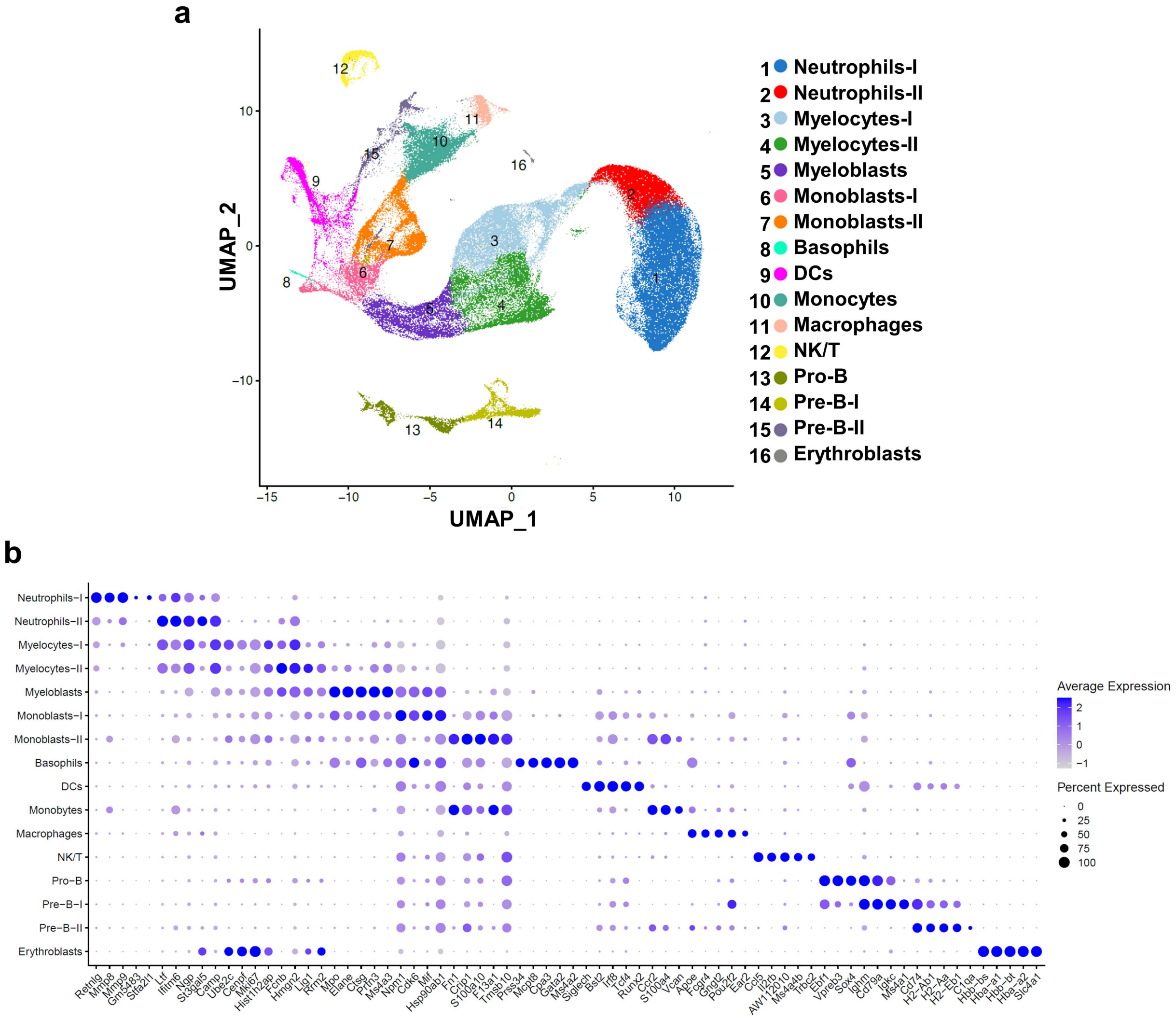

Supplementary Figure 10. scRNA-seq revealed tumor microenvironment modification after CD24-CAR-T cells treatment. (a) UMAP plot of whole BMMCs. Whole BMMCs were divided into 16 subclusters (Neutrophils-I, Neutrophils-II, Myelocytes-I, Myelocytes-II, Myeloblasts, Monoblasts-I, Monoblasts-II, Basophils, dendritic cells [DCs], Monocytes, Macrophages, Natural killer/ T cells [NK/T], Pro-B, Pre-B-I, Pre-B-II, Erythroblasts) (n = 3 per group). **(b)** Dot plot showed the main expression of marker genes across different immune cell types in mouse BM.

Supplementary Figure 11.

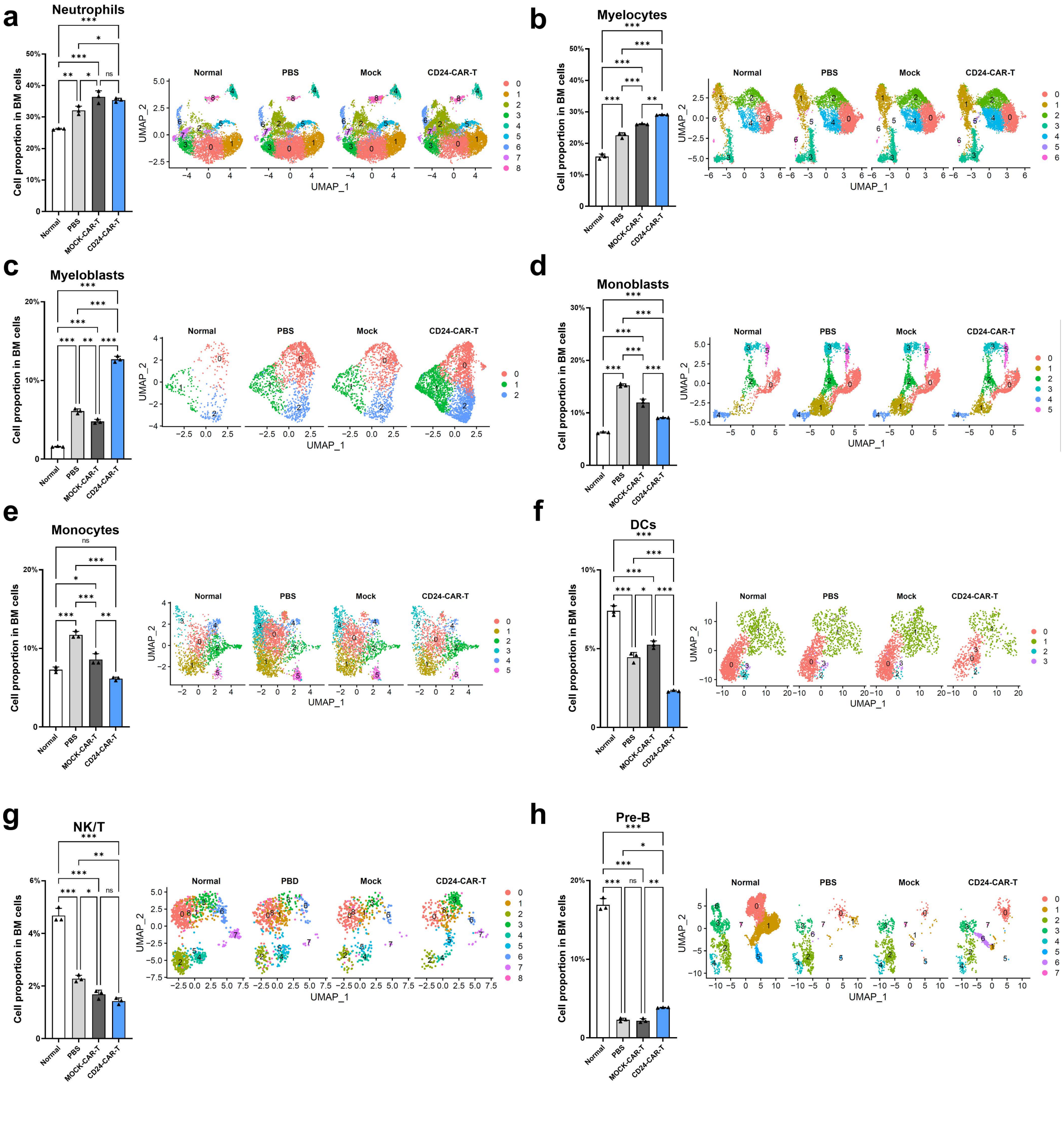

Supplementary Figure 11. Proportion of various cell types and sub-clusters. (a) Bar-views showed the proportion of neutrophils in whole BMMCs (left). UMAP plot of neutrophils subclusters (n = 3 mice per group). **(b)** Myelocytes (n = 3 mice per group). **(c)** Myeloblasts (n = 3 mice per group). **(d)** Monoblasts (n = 3 mice per group). **(e)** Monocytes (n = 3 mice per group). **(f)** DCs (n = 3 mice per group). **(g)** NK/T (n = 3 mice per group). **(h)** Pro-B (n = 3 mice per group). One-way ANOVA was used for statistical analysis. Data are presented as mean values +/- SD. $*P < .05$, $**P < .01$, $***P < .001$, ns = $P > .05$. Raw data is provided in the Source Data file. Exact P values for each comparison can be found in Supplementary Data 1.

Supplementary Figure 12.

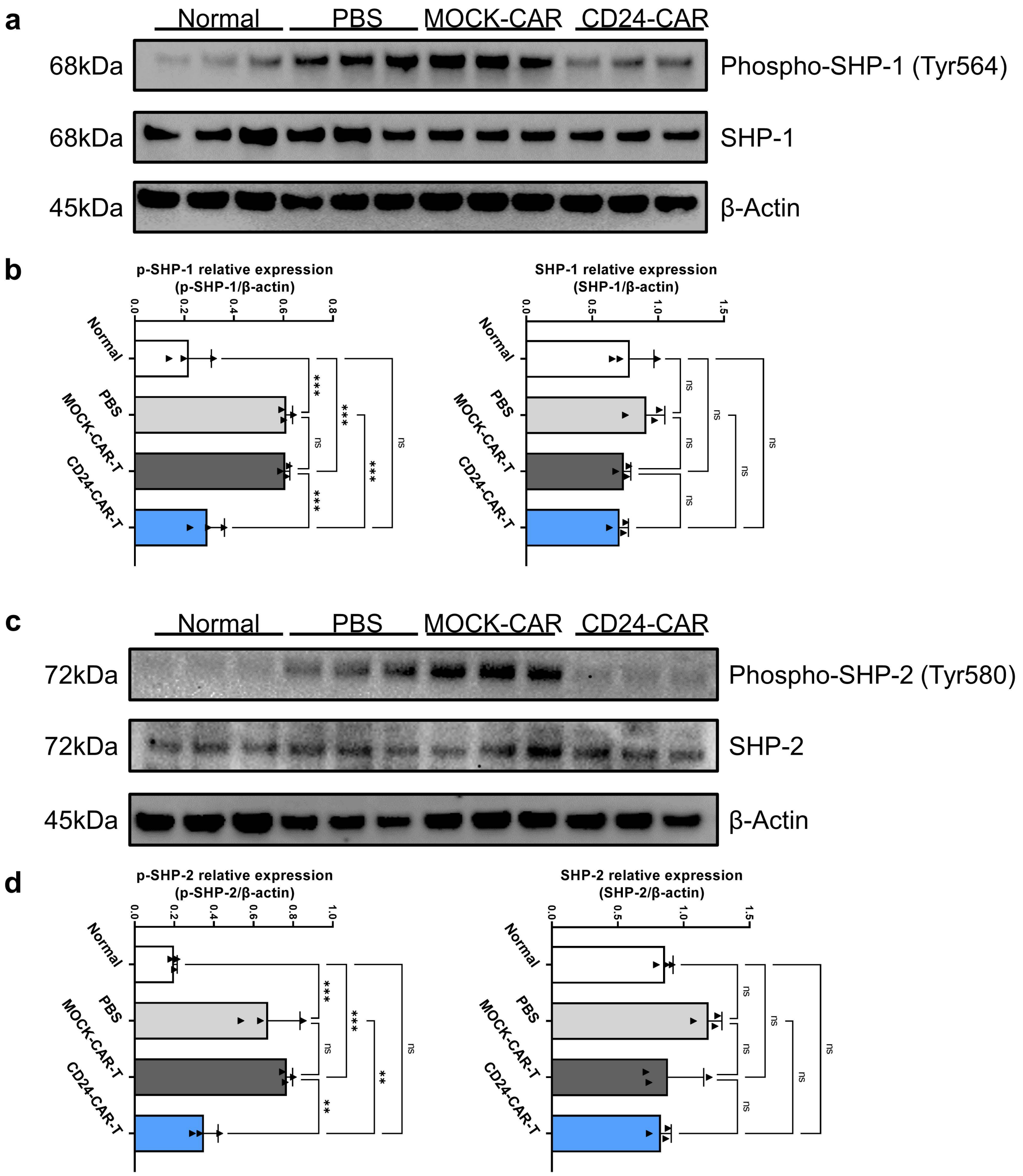

Supplementary Figure 12. The expression levels of SHP-1/2-related proteins in macrophages. (a) Western Blotting analysis of the protein expression levels of Phospho-SHP-1 (Tyr564), total SHP-1 and total β-actin (n = 3 independent experiments). **(b)** Relative expression of Phospho-SHP-1 (Tyr564) or total SHP-1 to β-actin. **(c)** Western Blotting analysis of the protein expression levels of Phospho-SHP-2 (Tyr580), total SHP-2 and total β-actin (n = 3 independent experiments). **(d)** Relative expression of Phospho-SHP-2 (Tyr580) or total SHP-2 to β-actin. The experiment was repeated twice with the same results. One-way ANOVA was used for statistical analysis. Data are presented as mean values +/- SD. $**P < .01$, $***P < .001$, ns = $P > .05$. Raw data is provided in the Source Data file. Exact P values for each comparison can be found in Supplementary Data 1.

Supplementary Figure 13.

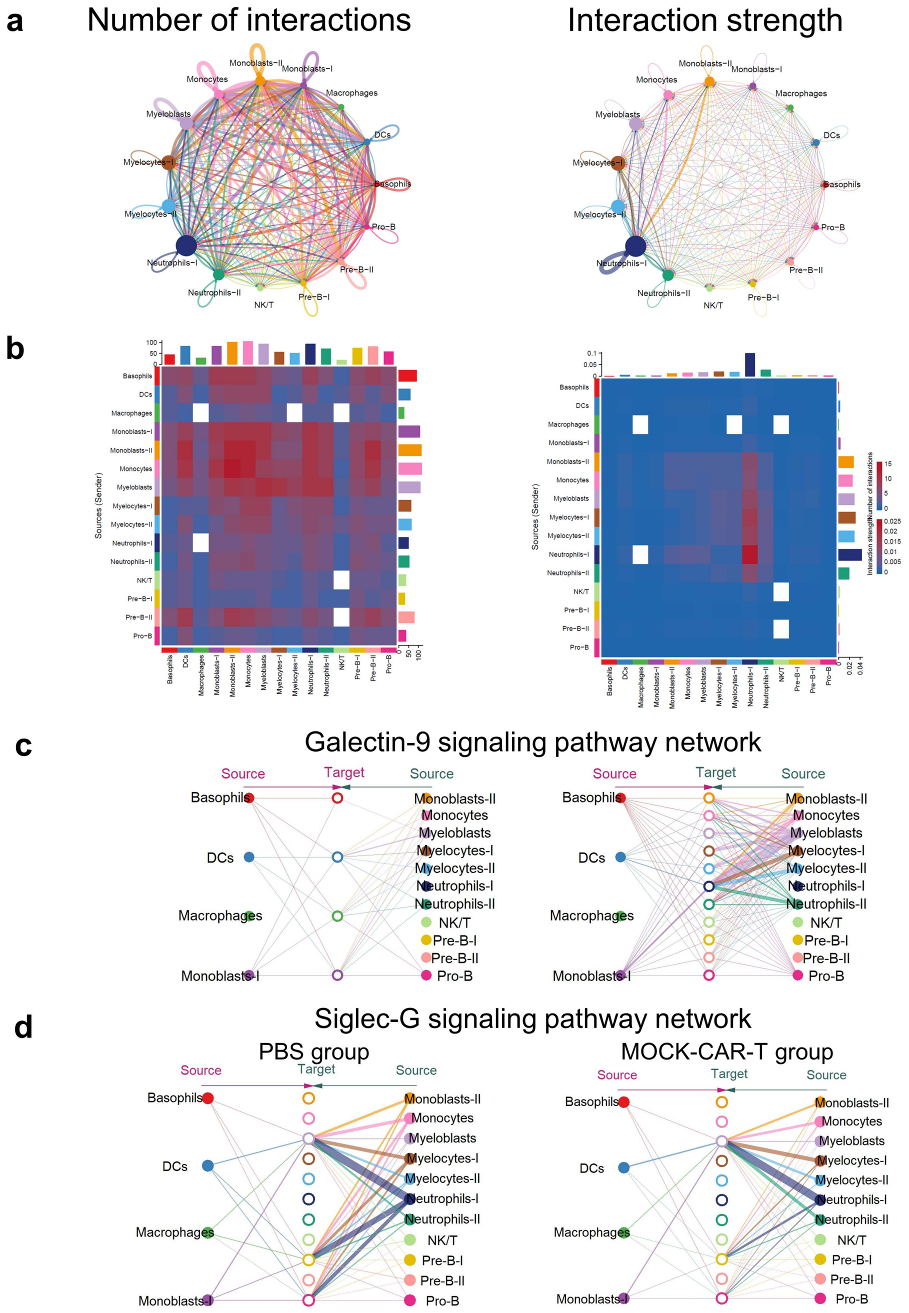

Supplementary Figure 13. Cell-cell communication analysis. (a) Circle plots of the number of interactions and interaction strength of CD24-CAR-T treatment group. **(b)** Heatmaps of the interactions and interaction strength of CD24-CAR-T treatment group. **(c)** Hierarchy plots showing the most involved communication signal in CD24-CAR-T treatment group. **(d)** Hierarchy plots showing the Siglec-G communication signal in PBS and MOCK-CAR-T treatment groups.

Supplementary Figure 14.

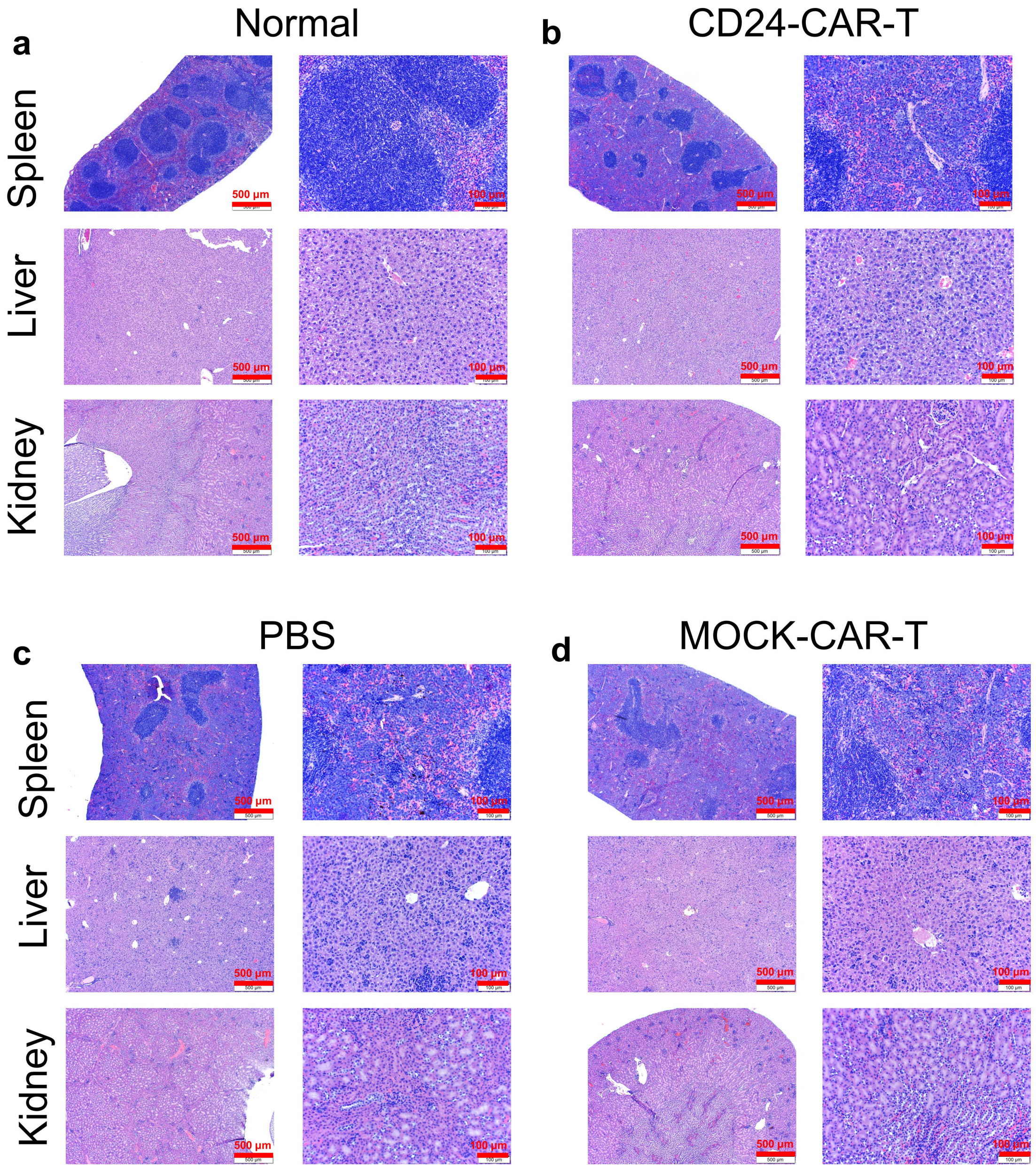

Supplementary Figure 14. CD24-CAR-T cells in the absence of toxicity. (a) Hematoxylin and eosin (H&E) stained spleen, liver, and kidney samples from normal C57BL/KaLwRij mice (n = 5 per group). **(b)** H&E stained spleen, liver, and kidney samples from MM-bearing mice treated with CD24-CAR-T cells (n = 5 per group). **(c)** H&E stained spleen, liver, and kidney samples from MM-bearing mice treated with PBS (n = 5 per group). **(d)** H&E stained spleen, liver, and kidney samples from MM-burden mice treated with MOCK-CAR-T cells (n = 5 per group). The experiment was repeated twice with the same results.

Supplementary Figure 15.

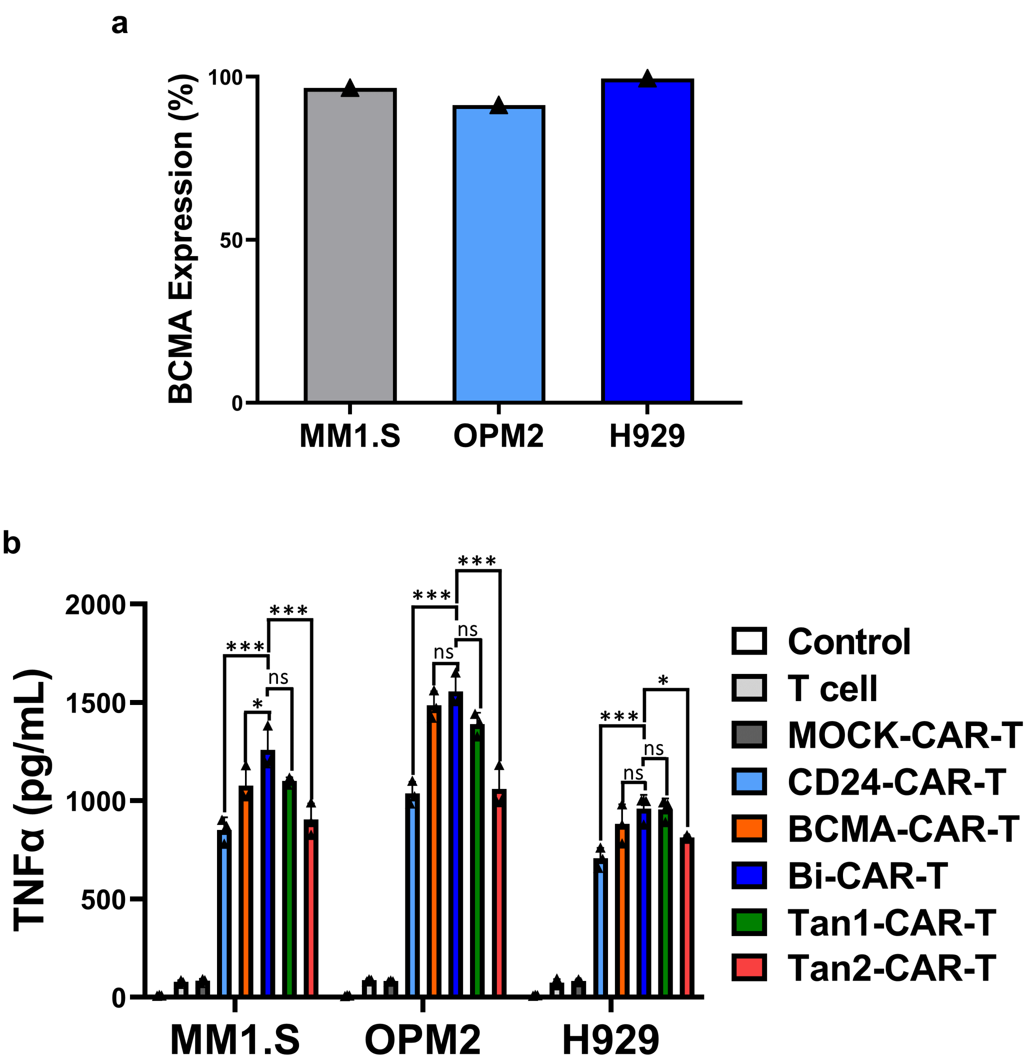

Supplementary Figure 15. BCMA expression in MM cell lines and bispecific CAR-T cells release TNF-α in vitro. (a) Bar plot showing expression levels of BCMA in MM1.S, OPM2, and H929 cell lines. **(b)**TNF-α concentrations on supernatants were detected when the E/T ratio was 5:1 after 24 hours of coculture (n = 3 independent experiments). One-way ANOVA was used for statistical analysis. Data are presented as mean values +/- SD. $*P < .05$, $***P < .001$, ns = $P > .05$. Raw data is provided in the Source Data file. Exact P values for each comparison can be found in Supplementary Data 1.

Supplementary Figure 16.

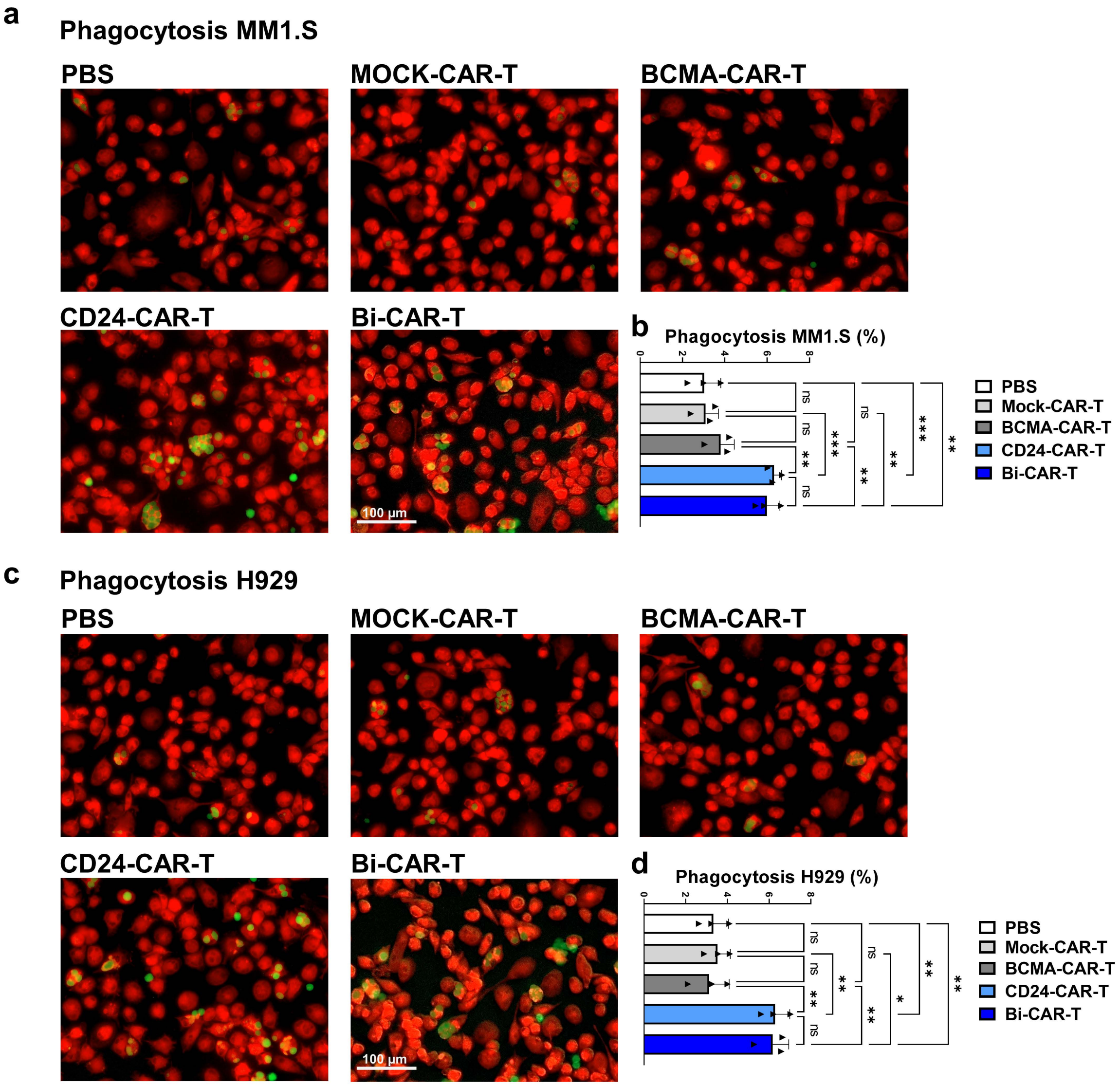

Supplementary Figure 16. CD24-CAR-T and Bi-CAR-T cells promote phagocytic clearance by macrophages in vitro. (a) Phagocytosis was performed by coculture of MM1.S cells that expressed GFP (green), DiD-stained macrophages (red), and CAR-T cells at a ratio of 2:1:1. After a 4-hour coculture, suspended cells were washed and detected. Fluorescent images of phagocytic clearance (n = 3 independent experiments). **(b)** Bar plot showing the percentage of MM1.S phagocytosis detected by flow cytometry analysis (n = 3 independent experiments). **(c)** Phagocytosis was performed by coculture of H929 cells (n = 3 independent experiments). **(d)** Bar plot showing the percentage of H929 phagocytosis detected by flow cytometry analysis (n = 3 independent experiments). The experiment was repeated twice with the same results. One-way ANOVA was used for statistical analysis. Data are presented as mean values +/- SD. $*P < .05$, $**P < .01$, $***P < .001$, ns = $P > .05$. Raw data is provided in the Source Data file. Exact P values for each comparison can be found in Supplementary Data 1.

Supplementary Figure 17.

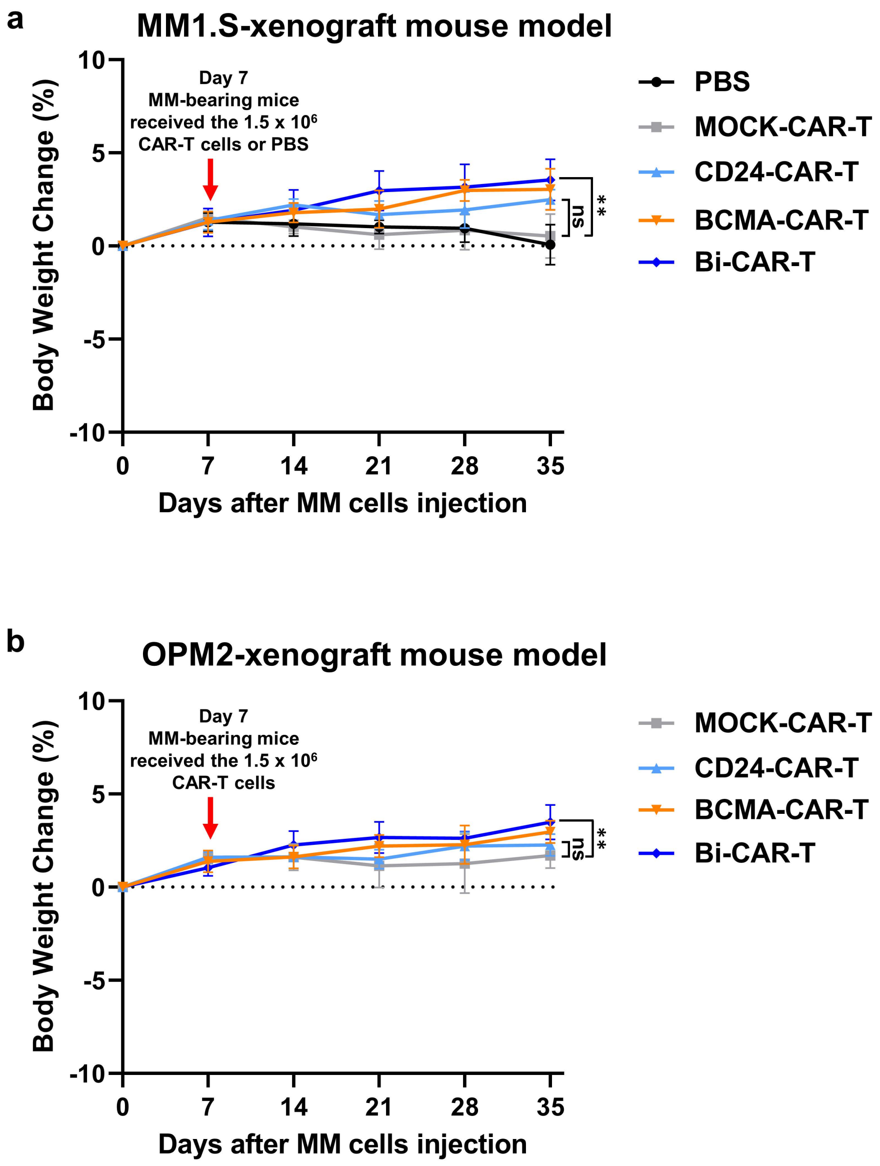

Supplementary Figure 17. Body weight changes in MM-xenograft mouse models. (a) Mouse weight normalized to day 0 for each group, from the day after MM1.S cells injection (n = 5 per group). **(b)** Mouse weight normalized to day 0 for each group, from the day after OPM2 cells injection (n = 5 per group). Data are presented as mean values +/- SD. One-way ANOVA was used for statistical analysis. ∗∗P < .01, ns = P > .05. Raw data is provided in the Source Data file. Exact P values for each comparison can be found in Supplementary Data 1.

Supplementary Figure 18.

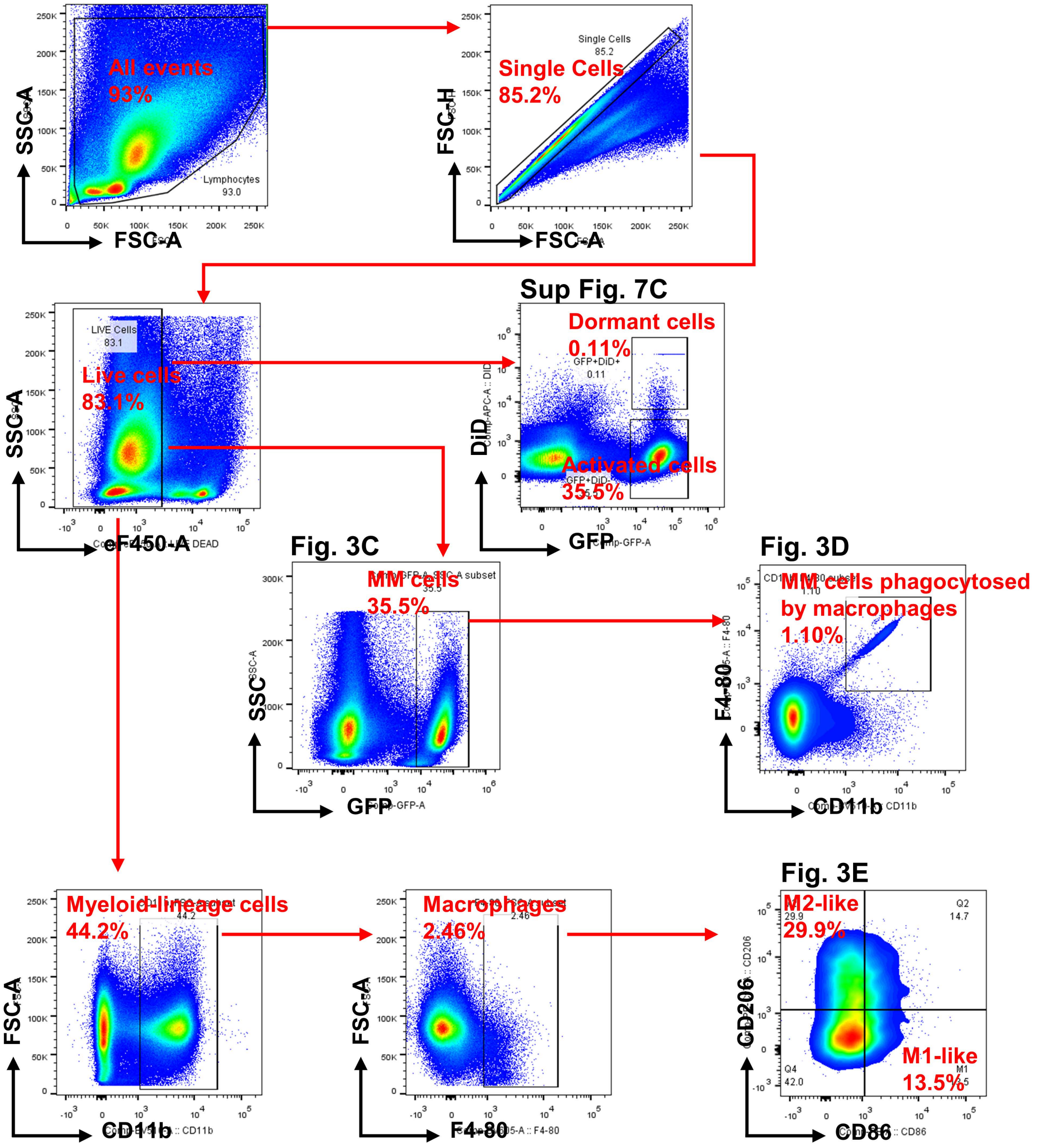

Supplementary Figure 18. Gating strategy for flow cytometry. After debris and doublet removal, myeloma cells were assessed as GFP$^+$ (Fig. 3C); dormant myeloma cells were assessed as GFP$^+$DiD$^+$ (Sup Fig. 7C); activated myeloma cells were assessed as GFP$^+$DiD$^-$ (Sup Fig. 7C); myeloma cells were phagocytosed by macrophages were assessed as GFP$^+$CD11b$^+$F4-80$^+$ (Fig. 3D); M1-like cells were assessed as CD11b$^+$F4-80$^+$CD86$^+$CD206$^-$; and M2-like cells were assessed as CD11b$^+$F4-80$^+$CD206$^+$CD86$^-$ (Fig. 3E).
