# Supplementary material for: Bispecific BCMA/CD24 CAR-T cells control multiple myeloma growth
Source: Nat Commun. 2024 Jan 19;15:615. doi: 10.1038/s41467-024-44873-4 (PMC10798961; doi:10.1038/s41467-024-44873-4)
Supplement: Supplementary file 3 — Description of Additional Supplementary Files [file 41467_2024_44873_MOESM3_ESM.pdf]

Description of Additional Supplementary Files Document

Supplementary Data 1 - Exact *P* values for indicated figures and comparisons.
