# Supplementary material for: Bispecific BCMA/CD24 CAR-T cells control multiple myeloma growth
Source: Nat Commun. 2024 Jan 19;15:615. doi: 10.1038/s41467-024-44873-4 (PMC10798961; doi:10.1038/s41467-024-44873-4)
Supplement: Supplementary file 5 — Reporting Summary [file 41467_2024_44873_MOESM5_ESM.pdf]

nature portfolio

Corresponding author(s): Fenghuang Zhan

Last updated by author(s): Jan 7, 2024

Reporting Summary

Nature Portfolio wishes to improve the reproducibility of the work that we publish. This form provides structure for consistency and transparency in reporting. For further information on Nature Portfolio policies, see our Editorial Policies and the Editorial Policy Checklist.

Statistics

For all statistical analyses, confirm that the following items are present in the figure legend, table legend, main text, or Methods section.

n/a	Confirmed	
☐	☒	The exact sample size (*n*) for each experimental group/condition, given as a discrete number and unit of measurement
☐	☒	A statement on whether measurements were taken from distinct samples or whether the same sample was measured repeatedly
☐	☒	The statistical test(s) used AND whether they are one- or two-sided<br>*Only common tests should be described solely by name; describe more complex techniques in the Methods section.*
☒	☐	A description of all covariates tested
☒	☐	A description of any assumptions or corrections, such as tests of normality and adjustment for multiple comparisons
☐	☒	A full description of the statistical parameters including central tendency (e.g. means) or other basic estimates (e.g. regression coefficient) AND variation (e.g. standard deviation) or associated estimates of uncertainty (e.g. confidence intervals)
☐	☒	For null hypothesis testing, the test statistic (e.g. *F*, *t*, *r*) with confidence intervals, effect sizes, degrees of freedom and *P* value noted<br>*Give P values as exact values whenever suitable.*
☒	☐	For Bayesian analysis, information on the choice of priors and Markov chain Monte Carlo settings
☒	☐	For hierarchical and complex designs, identification of the appropriate level for tests and full reporting of outcomes
☒	☐	Estimates of effect sizes (e.g. Cohen's *d*, Pearson's *r*), indicating how they were calculated

Our web collection on statistics for biologists contains articles on many of the points above.

Software and code

Policy information about availability of computer code

Data collection	FACS Diva software v7 (BD); Fluorescence-based killing assays were measured using BioTek Cytation 5 Cell Imaging Multimode Reader software (Agilent Technologies); Fluorescence images were acquired using ZEISS Axio Observer fluorescence microscope software ZEN3.5 (ZEISS); In vivo bioluminescence images were acquired using IVIS living image version 4.4 software (Caliper Life Sciences); Single Cell RNA sequencing using the Chromium platform from 10x Genomics 3’ v3.1 (10x Genomics); No commercial, open source, or custom code was used for this study.
Data analysis	Flowjo V10 (BD), GraphPad Prism 9 (GraphPad Software Inc.) were used for flow cytometry analysis.<br>GraphPad Prism 9 (GraphPad Software Inc.) were used for fluorescence-based killing assays.<br>IVIS living image software and GraphPad Prism 9 (GraphPad Software Inc.) were used for bioluminescence images assays.<br>Fluorescence microscope software ZEN3.5 (ZEISS) and GraphPad Prism 9 (GraphPad Software Inc.) were used for fluorescence images assays.<br>Single-cell emulsions were generated with the Chromium Next GEM Chip G Single Cell Kit (10X Genomics) and the Chromium Next GEM Single Cell 3’ v3.1 Kit (10X Genomics) following the standard protocol. Libraries were assessed for mass concentration with the Qubit 1X dsDNA High Sensitivity Assay Kit (Thermo Fisher Scientific). Library fragment size was assessed with the High Sensitivity NGS Fragment Analysis Kit (Agilent) on the Fragment Analyzer System (Agilent). Libraries were functionally validated with the KAPA Library Quantification Kit (Roche). Initial low-pass “surveillance” sequencing was performed on a NovaSeq SP 100-cycle Flow Cell (Illumina) and data was assessed with the Cell Ranger count (10X Genomics) output.<br>The raw scRNA-seq data were loaded into R through the Seurat V4 package. The Seurat's integration workflows were used for quality control. First, cells exhibiting low-complexity libraries, indicating detection of transcripts aligned to fewer than 200 genes, potentially representing dying or apoptotic cells (more than 10% of unique molecular identifiers stemming from mitochondrial genes), as well as cells with high-complexity libraries (with detected transcripts aligned to more than 7,000 genes), were excluded. We used DoubletFinder function to identify potential doublets within our dataset. Essentially, a doublet is characterized as a single-cell library that represents more than one cell. Upon

closer inspection of certain known markers, it was observed that the implicated cluster comprises doublets of more than one cell type, as no cell type is recognized for robustly expressing both markers simultaneously. Doublets were individually removed from each sample, employing an anticipated doublet rate of 0.05. Subsequently, the cell expression matrix of each sample underwent normalization using the NormalizeData function with default parameters. Following this, the FindVariableFeatures function, with default parameters, was employed to identify highly variable genes (HVGs) within each normalized matrix. The SelectIntegrationFeatures function was then utilized, specifying nfeatures=2,000, to select genes for the integration of multiple samples. To mitigate the impact of the cell cycle on data integration, we excluded cell-cycle-related genes from the gene set. Sequentially, the RunPCA and ScaleData functions were applied with the parameter features set to these selected genes. Subsequent to scaling the matrix for each dataset, PCA was conducted. To reduce batch effects, we applied the "anchor" integration method (functions FindIntegrationAnchors and IntegrateData). We employed the FindIntegrationAnchors function, specifying reduction='rpca,' to identify a set of anchors between all matrices. These anchors were used to integrate the matrices through the IntegrateData function with parameter dims=1:50. Finally, the ScaleData function was applied to scale the integrated matrix using default parameters. Then the nitration results were used as input for clustering with the Louvain algorithm with multilevel refinement and UMAP. The gene-specific markers for each cluster were determined using the FindMarkersAll function with Model-based Analysis of Single-cell Transcriptomics (MAST) test statistics. Subsequently, the top 20 gene-specific markers were input into the CellMarker 2.0 cell annotation tool to obtain automatic annotations. Then, cluster annotations were generated through a combination of automatic annotation and manual annotation based on relevant studies. Gene-set enrichment analysis of these marker genes was carried out for kyoto encyclopedia of genes and genomes (KEGG) pathway analysis. Differentially expressed genes (DEGs) were denoted as statistically significant for the false discovery rate (FDR) less than 0.05 with a fold change exceeding 1.2. No new algorithms were developed for this manuscript. All original code has been deposited to GitHub (DOI: 10.5281/zenodo.10014735).

For manuscripts utilizing custom algorithms or software that are central to the research but not yet described in published literature, software must be made available to editors and reviewers. We strongly encourage code deposition in a community repository (e.g. GitHub). See the Nature Portfolio guidelines for submitting code & software for further information.

Data

Policy information about availability of data

All manuscripts must include a data availability statement. This statement should provide the following information, where applicable:

- Accession codes, unique identifiers, or web links for publicly available datasets
- A description of any restrictions on data availability
- For clinical datasets or third party data, please ensure that the statement adheres to our policy

The mouse single-cell RNA sequencing publicly available data used in this study are available in the Gene Expression Omnibus (GEO) database under accession code GSE226956 (https://www.ncbi.nlm.nih.gov/geo/query/acc.cgi?acc=GSE226956). The human single-cell RNA sequencing publicly available data used in this study are available in the GEO database under accession code GSE210079 (https://www.ncbi.nlm.nih.gov/geo/query/acc.cgi?acc=GSE210079). The remaining data are available within the Article, Supplementary Information, or Source Data file. Source data are provided with this paper.

Research involving human participants, their data, or biological material

Policy information about studies with human participants or human data. See also policy information about sex, gender (identity/presentation), and sexual orientation and race, ethnicity and racism.

Reporting on sex and gender	Human BMMCs were obtained from University of Arkansas for Medical Sciences (UAMS) Myeloma Center Tissue Biorepository and Procurement Core. This study was composed of 56 females and 70 males. Sex was not used as a variable in study analyses, and study findings are not specific to one sex.
Reporting on race, ethnicity, or other socially relevant groupings	Human BMMCs were obtained from University of Arkansas for Medical Sciences (UAMS) Myeloma Center Tissue Biorepository and Procurement Core. This study was composed of 33 African Americans and 93 Caucasian. Race was not used as a variable in study analyses, and study findings are not specific to one race, ethnicity, or other socially relevant groupings.
Population characteristics	Monoclonal gammopathy of undetermined significance (MGUS) and smoldering multiple myeloma (SMM), newly diagnosed MM, and relapsed/refractory MM (RRMM)(IRB 261817 and 261821). Female and male patients with an average age of 62 +/-8.8 (average +/- SD). Participants were recruited to the Myeloma Center Tissue Biorepository and Procurement Core protocols IRB 261817 and 261821 based on having a diagnosis of MGUS, SMM or MM and being interested in donating a research bone marrow sample to UAMS. All participants provided written informed consent for sample procurement in accordance with the Declaration of Helsinki.
Recruitment	Participants were recruited to the Myeloma Center Tissue Biorepository and Procurement Core protocols IRB 261817 and 261821 based on having a diagnosis of MGUS, SMM or MM and being interested in donating a research bone marrow sample to UAMS. All participants provided written informed consent for sample procurement in accordance with the Declaration of Helsinki. Human patient bone marrow samples were collected with written informed consent and processed at University of Arkansas for Medical Sciences (UAMS) Myeloma Center Tissue Biorepository and Procurement Core, in accordance with recognized ethical guidelines, under an approved IRB 261817 and 261821. All the samples were de-identified. All participants provided informed consent and did not receive any compensation. The recruitment process did not involve any selection bias.
Ethics oversight	Human BMMCs were obtained from University of Arkansas for Medical Sciences (UAMS) Myeloma Center Tissue Biorepository and Procurement Core. The UAMS institutional review board approved these research studies (IRB 261817 and 261821). All participants provided written informed consent for sample procurement in accordance with the Declaration of Helsinki.

Note that full information on the approval of the study protocol must also be provided in the manuscript.

Field-specific reporting

Please select the one below that is the best fit for your research. If you are not sure, read the appropriate sections before making your selection.

☒ Life sciences ☐ Behavioural & social sciences ☐ Ecological, evolutionary & environmental sciences

For a reference copy of the document with all sections, see nature.com/documents/nr-reporting-summary-flat.pdf

Life sciences study design

All studies must disclose on these points even when the disclosure is negative.

Sample size	Sample sizes for in vivo experiments, e.g. treatment cohorts, were used on the basis of ensuring results obtained were of a representable quantity. A population range of 5 mice per group was used to ensure statistical power. For in vitro studies, sample size of at least 3 was used in each experiment. Sample size and number of independent experiments are stated in the figure legends.
Data exclusions	The count table was loaded into R through the Seurat V4 package for further analysis. The cells that have fewer than 500 genes, greater than 5,000 genes, and more than 10% of unique molecular identifiers stemming from mitochondrial genes were discarded from the analysis.
Replication	All experiments were reliably reproduce. All experiments were performed independently at least two times. Single Cell RNA sequencing samples were pooled from 3 mice in each group.
Randomization	Once mice were confirmed to carry tumors, they were randomized into different treatment cages.<br>In vitro experiments were randomly assigned to different groups.
Blinding	For animal studies, tumor burden, survival measurements, and tumor cell proportions were carried out by an operator who was blinded to treatment groups. In vitro experiments were not blinded due to the non-biased collection of data using computer software.<br>The function analysis was blinded by de-identifying samples. Investigators were blinded to group allocation during data collection.

Reporting for specific materials, systems and methods

We require information from authors about some types of materials, experimental systems and methods used in many studies. Here, indicate whether each material, system or method listed is relevant to your study. If you are not sure if a list item applies to your research, read the appropriate section before selecting a response.

Materials & experimental systems

n/a	Involved in the study	
☐	☒	Antibodies
☐	☒	Eukaryotic cell lines
☒	☐	Palaeontology and archaeology
☐	☒	Animals and other organisms
☒	☐	Clinical data
☒	☐	Dual use research of concern
☒	☐	Plants

Methods

n/a	Involved in the study	
☒	☐	ChIP-seq
☐	☒	Flow cytometry
☒	☐	MRI-based neuroimaging

Antibodies

Antibodies used

The flow cytometry antibodies were used:
APC anti-human CD24 Antibody (Biolegend Cat#: 311118 Clone:ML5) 1:200 dilution
APC/Cyanine7 anti-human CD24 Antibody (Biolegend Cat#: 311131 Clone:ML5) 1:200 dilution
FITC anti-human CD138 (Syndecan-1) Antibody (Biolegend Cat#: 352304 Clone:DL-101) 1:200 dilution
PE anti-human CD4 Antibody (Biolegend Cat#: 357404 Clone:A161A1) 1:200 dilution
APC anti-human CD8 Antibody (Biolegend Cat#: 344722 Clone:SK1) 1:200 dilution
APC/Cyanine7 anti-human CD45RO Antibody (Biolegend Cat#: 304227 Clone:UCHL1) 1:200 dilution
FITC anti-human CD62L Antibody (Biolegend Cat#: 304838 Clone:DREG-56) 1:200 dilution
APC/Cyanine7 anti-human CD3 Antibody (Biolegend Cat#: 300318 Clone:HIT3a) 1:200 dilution
PE anti-human CD34 Antibody (Invitrogen Cat#: MA1-10205 Clone:QBEND/10) 1:200 dilution
FITC Anti-Human CD69 Antibody (BD Cat#: 555530 Clone:FN50) 1:200 dilution
Anti-human CD24 Antibody (InVivo BioTech Cat#: AK208.2/06B.1 Clone: SWA11) 1:200 dilution
BV510 anti-mouse/human CD11b Antibody (Biolegend Cat#: 101263 Clone: M1/70) 1:200 dilution
BV605 Rat Anti-Mouse F4/80 Antibody (BD Cat#: 743281 Clone:T45-2342) 1:200 dilution
PE anti-mouse CD86 Antibody (Biolegend Cat#: 105008 Clone:GL-1) 1:200 dilution
PE/Cyanine7 anti-mouse CD206 (MMR) Antibody (Biolegend Cat#: 141720 Clone:C068C2) 1:200 dilution
PE Rat Anti-Mouse CD24 Antibody (BD Cat#: 553262 Clone:M1/69) 1:200 dilution

The Western Blotting antibodies were used:

	SHP-1 Antibody (Cell Signaling Technology, catalog # 3759, clone # C14H6) 1:1000 dilution<br>SHP-2 Antibody (Cell Signaling Technology, catalog # 3397, clone # D50F2) 1:1000 dilution<br>Phospho-SHP-1 Antibody (Tyr564) (Cell Signaling Technology, catalog # 8849, clone # D11G5) 1:1000 dilution<br>Phospho-SHP-2 Antibody (Tyr580) (Cell Signaling Technology, catalog # 5431, clone # D66F10) 1:1000 dilution<br>β-Actin Antibody (Cell Signaling Technology, catalog # 4967) 1:1000 dilution<br>Anti-rabbit IgG, HRP-linked antibody (Cell Signaling Technology, catalog # 7074) 1:1000 dilution
Validation	The flow cytometry antibodies' validation:<br>APC anti-human CD24 Antibody, tested by flow cytometry on human peripheral blood lymphocytes (manufacturer)<br>APC/Cyanine7 anti-human CD24 Antibody, tested by flow cytometry on human peripheral blood lymphocytes (manufacturer)<br>FITC anti-human CD138 (Syndecan-1) Antibody, tested by flow cytometry on human myeloma cell line U266 (manufacturer)<br>PE anti-human CD4 Antibody, tested by flow cytometry on human peripheral blood lymphocytes (manufacturer)<br>APC anti-human CD8 Antibody, tested by flow cytometry on human peripheral blood lymphocytes (manufacturer)<br>APC/Cyanine7 anti-human CD45RO Antibody, tested by flow cytometry on human peripheral blood lymphocytes (manufacturer)<br>FITC anti-human CD62L Antibody, tested by flow cytometry on human peripheral blood lymphocytes (manufacturer)<br>APC/Cyanine7 anti-human CD3 Antibody, tested by flow cytometry on human peripheral blood lymphocytes (manufacturer)<br>PE anti-human CD34 Antibody, tested by flow cytometry on human peripheral whole blood (manufacturer)<br>FITC Anti-Human CD69 Antibody, tested by flow cytometry on human PBMC (manufacturer)<br>Anti-human CD24 Antibody, tested by flow cytometry on human PBMC (manufacturer)<br>BV510 anti-mouse/human CD11b Antibody, tested by flow cytometry on C57BL/6 mouse bone marrow cells (manufacturer)<br>BV605 Rat Anti-Mouse F4/80 Antibody, tested by flow cytometry on C57BL/6 mouse splenic leucocytes (manufacturer)<br>PE anti-mouse CD86 Antibody, tested by flow cytometry on LPS (3-day) stimulated BALB/c mouse splenocytes (manufacturer)<br>PE/Cyanine7 anti-mouse CD206 (MMR) Antibody, tested by flow cytometry on Thioglycollate-elicited BALB/c mouse peritoneal macrophages (manufacturer)<br>PE Rat Anti-Mouse CD24 Antibody, tested by flow cytometry on BALB/c thymocytes (manufacturer)<br><br>The Western Blotting antibodies' validation:<br>SHP-1 Antibody, tested by Western blot on Jurkat, 32D and BaF3 cells (manufacturer)<br>SHP-2 Antibody, tested by Western blot on HeLa, 293, COS and NIH/3T3 cells (manufacturer)<br>Phospho-SHP-1 Antibody, tested by Western blot on Jurkat and JCaM1.6 cells (manufacturer)<br>Phospho-SHP-2 Antibody, tested by Western blot on C6 cells (manufacturer)<br>β-Actin Antibody, tested by Western blot on JHeLa, C2C12, C6, COS, and MvLu cells (manufacturer)<br>Anti-rabbit IgG, HRP-linked antibody, thoroughly validated with Cell Signaling Technology primary antibodies (manufacturer)

Eukaryotic cell lines

Policy information about cell lines and Sex and Gender in Research

Cell line source(s)	Human MM1.S (ATCC#: CRL-2974), RPMI 8226 (ATCC#: CCL-155) cells were purchased from ATCC. Mouse 5TGM1 cells were purchased from Harlan Laboratory Inc (Reference#: 799). Human OPM2, MM1-144, KMS-34, FR4, XG-7, MMM1, KAS-6/1, H929, Delta 47, OCI-MY5, AMO1, JIM3, VPC-6, KMS12-PE, ARP-1, KMS-11, KMS12BM, JK-6L cells were generously provided by Dr. Siegfried Janz (Medical College of Wisconsin, Milwaukee, WI, USA).
Authentication	All cell lines were authenticated by STR profiling prior to use. All experiments were performed using aliquots of identified cell lines.
Mycoplasma contamination	All cell lines tested negative for mycoplasma.
Commonly misidentified lines<br>(See ICLAC register)	No commonly misidentified cell lines were used in this study.

Animals and other research organisms

Policy information about studies involving animals; ARRIVE guidelines recommended for reporting animal research, and Sex and Gender in Research

Laboratory animals	Mus musculus, C57BL/KalwRijHsd mice were purchased from the Harlan Laboratory (https://www.envigo.com/model/c57bl-kalwrijhsd). Mus musculus, NOD.Cg-Prkdcscid Il2rgtm1Wjl/SzJ mice were purchased from the Jackson Laboratory (https://www.jax.org/strain/005557). Mice used in the experiments are sex mixed at the age of 8-10 weeks. Mice were housed under social conditions (2-5 mice per cage) on a standard 12-hour dark/12-h light cycle, ambient temperature 21 °C ± 1 °C, and humidity 50% ± 10%. All mice were housed in a pathogen-free animal facility with standard food and water.
Wild animals	This study did not involve wild animals.
Reporting on sex	This study did not only use one sex.
Field-collected samples	This study did not involve field-collected samples.
Ethics oversight	All animal protocols undergo a strict approval process with the Institutional Animal Care and Use Committee (IACUC) at University of Arkansas for Medical Sciences. The protocol number is IACUC 3997.

Note that full information on the approval of the study protocol must also be provided in the manuscript.

Flow Cytometry

Plots

Confirm that:

☒ The axis labels state the marker and fluorochrome used (e.g. CD4-FITC).

☒ The axis scales are clearly visible. Include numbers along axes only for bottom left plot of group (a 'group' is an analysis of identical markers).

☒ All plots are contour plots with outliers or pseudocolor plots.

☒ A numerical value for number of cells or percentage (with statistics) is provided.

Methodology

Sample preparation	Sample preparation was performed accordingly to the guidelines for Cell Surface Flow Cytometry Staining Protocol: https://www.biolegend.com/en-us/protocols/cell-surface-flow-cytometry-staining-protocol
Instrument	BD LSRFortessa Flow Cytometer and BD FACSverse Flow Cytometer
Software	FACS Diva software v7 (BD)
Cell population abundance	Purity of cell population checked by flow cytometry
Gating strategy	or all experiments, FSC-A/SSC-A gates of the starting cell population were used to discriminate between viable cells and cell debris. Singlet and doublet cells were discriminated using FSC-H/FSC-A gating. Live cells were selected by gating 7-AAD (Viability Staining Solution) negative cells. Then gating was depended on clear distinguish cell population, and also by comparing to control staining samples. T cells memory phenotypes were assigned according to CD62L and CD45RO expression within the CAR-T cell population as follows: stem cell memory (CD45RO-/CD62L+), central memory (CD45RO+/CD62L+), effector memory (CD45RO+/CD62L-), effector cells (CD45RO-/CD62L-).

☒ Tick this box to confirm that a figure exemplifying the gating strategy is provided in the Supplementary Information.
